# Biomechanical Variables Associated with the First 20 m of the Start Phase in Speed Skaters

**DOI:** 10.3390/sports14080336

**Published:** 2026-08-04

**Authors:** Javier Gaviria-Chavarro, Miguel Ángel Gómez-García, Claudia F. Giraldo-Jiménez

**Affiliations:** 1 Doctoral Program in Applied Sciences, Faculty of Basic Sciences, Universidad Santiago de Cali, Cali 760011, Colombia; 2 Faculty of Sport and Education Sciences, Institución Universitaria Escuela Nacional del Deporte, Cali 760033, Colombia; mangelgomez@endeporte.edu.co; 3 Physiotherapy Program, Faculty of Health, Universidad Santiago de Cali, Cali 760011, Colombia; claudia.giraldo00@usc.edu.co

**Keywords:** Bayesian analysis, speed skating, kinematics, biomechanics, video analysis

## Abstract

This exploratory study examined segmental biomechanical variables associated with 20 m start performance in 24 juvenile inline speed skaters (14–16 years). Athletes performed three maximal 20 m starts; the best time was recorded with photoelectric timing gates, and the corresponding trial was analyzed from two-dimensional video using Tracker. Segmental kinematics and indirect inertial estimates were derived using De Leva-adjusted anthropometric parameters. Candidate predictors were selected with LASSO, and the complete preprocessing and selection pipeline was evaluated using nested leave-one-out cross-validation. Post-selection ordinary least squares, bootstrap, and Bayesian models were used as secondary analyses of coefficient sensitivity and uncertainty. LASSO retained forearm segmental inertial force estimate, thigh velocity, forearm velocity, thigh angular velocity, and torso angular acceleration. The nested pipeline achieved RMSE = 0.12 s, MAE = 0.08 s, and R^2^_pred = 0.485. Greater thigh velocity, forearm inertial force estimate, thigh angular velocity, and torso angular acceleration were associated with shorter times, whereas greater forearm velocity was associated with longer times. These findings identify candidate within-sample associations, not stable biomechanical determinants. Because of the small sample size, cross-sectional design, indirect two-dimensional measurements, absent reliability analysis, and lack of external validation, the variables should be used only for hypothesis generation and provisional monitoring.

## 1. Introduction

Speed skating comprises two primary competitive disciplines: long-distance events, which predominantly rely on aerobic endurance, and sprint events, which primarily depend on the phosphagen and glycolytic energy systems. These physiological demands are determined by race distance and the performance requirements associated with each event [1]. The start phase is considered a critical determinant of skating performance, as previous research has shown that balance and muscle power explain up to 78% of the variance in start performance, highlighting the importance of these physical capacities during the initial acceleration phase [2,3,4].

Athletes who execute an effective start gain an immediate competitive advantage because acceleration during the initial meters substantially influences overall race performance. From a biomechanical perspective, start performance depends on the coordinated interaction of multiple body segments that generate, transmit, and regulate mechanical forces throughout the acceleration phase. The lower limbs constitute the primary source of propulsive force through their interaction with the skating surface, whereas the trunk contributes to postural stabilization and facilitates the transmission of mechanical forces between the lower and upper extremities. Concurrently, upper-limb motion contributes to whole-body coordination by regulating angular momentum and optimizing movement efficiency during skating. Consequently, biomechanical variables describing the kinematic and mechanical behavior of these body segments provide a theoretical basis for identifying predictors of start performance and understanding the mechanical factors underlying skating acceleration [5,6,7,8].

Beyond the contribution of the lower limbs, trunk, and upper limbs, efficient sports performance also depends on the integration of the cervical region within the whole-body neuromuscular system. The cervical spine contributes to head stabilization, postural control, sensorimotor integration, and the coordination of body segments during dynamic movements, thereby facilitating the transmission and regulation of mechanical forces throughout the kinetic chain. This integrated perspective is consistent with the kinetic chain concept, whereby efficient force production and movement execution emerge from coordinated interactions among body segments rather than from isolated regional actions [9]. In addition, the stomatognathic and cervical systems exhibit close biomechanical and neurophysiological interactions through shared muscular, fascial, and neural connections, suggesting that alterations in one system may influence cervical mobility and motor control. Nevertheless, current evidence regarding the extent to which these interactions affect sports performance remains inconclusive and the subject of scientific debate [10]. Therefore, cervical function should be considered a complementary component of whole-body neuromuscular coordination rather than an isolated determinant of athletic performance.

Executing human movement requires precise coordination of multiple muscle groups. When this movement is performed using an external implement—such as skates, a ball, or a racket—the motor action must adapt to the characteristics of the implement and the specific demands of the technical gesture. Sports such as speed skating are characterized by the development of highly specialized motor patterns. In this regard, understanding how biomechanical variables influence movement is essential [11]. In speed skating, this is particularly relevant during the first 20 m of the start phase. When executed efficiently, this phase is considered a key component of performance [3,7].

To analyze this start phase, both the kinematics and kinetics of movement must be examined, as this allows the technique to be studied from a quantitative perspective and enables the identification of the variables most closely related to performance in the first 20 m. In sprint events, stride frequency is a key factor, whereas in endurance efforts, stride length becomes more relevant [12]. From a muscle activation perspective, the posterior chain plays a central role: hip extension and abduction favor the participation of the gluteus maximus, gluteus medius, vastus lateralis, biceps femoris, and rectus femoris during propulsion at high velocities [13]. These findings provide a biomechanical rationale to explain the importance of the first 20 m in competitive performance.

However, conventional descriptive and inferential analyses do not necessarily provide an adequate interpretation of the complex relationships among biomechanical variables. Therefore, it is important to examine biomechanical variables associated with performance to gain a deeper understanding of the kinematic behavior of body segments during the starting technique. Wu et al. [14] highlighted the limited evidence available on skating biomechanics, particularly regarding the identification of biomechanical determinants of performance. They argued that previous studies have predominantly focused on descriptive assessments of segmental kinematics (e.g., angular position and joint motion), whereas relatively few have employed analytical or predictive approaches to identify the biomechanical variables associated with elite performance. In addition, the available evidence indicates that, between 5 and 40 m, variables such as velocity, maximum mechanical power, and force production are commonly evaluated through indicators such as the rate of force development and the force–velocity–power curve [7]. Video analysis is also a useful tool for examining technique, preventing injuries, measuring performance, and optimizing technical aspects of movement [15,16].

In this context, biomechanics applied to skating uses various analytical tools. Among them, three-dimensional models have been developed to study technique and quantify the joint angles of different body segments [17]. In skating, these models have shown that considerable angular displacement of the knee and hip occurs during the gliding phase [18]. Similarly, ground reaction forces are a key component that should be incorporated when analyzing movement [7,19]. In turn, other devices, such as inertial sensors, have shown high accuracy and reliability in obtaining data related to motor performance [20].

The literature review also identified several knowledge gaps. First, there is a lack of methodological approaches incorporating the Tracker software for biomechanical analysis in sports settings, particularly in speed skating. Furthermore, only a limited number of studies have estimated body-segment kinematic variables using Zatsiorsky’s cadaver-based anthropometric model, as modified by De Leva [21]. In addition, Bayesian statistical approaches remain largely unexplored in skating biomechanics research, despite their potential to provide robust probabilistic inference and improve the identification of biomechanical determinants of performance [22]. These limitations highlight important opportunities for future research in the field of applied skating biomechanics.

Exploratory analysis of body-segment kinematic variables during the start phase may help identify candidate features associated with performance and generate hypotheses for future investigation [23]. Given the cross-sectional design, the small sample size, and the indirect two-dimensional estimates, these features should not be interpreted as established individual deficiencies or as immediate targets for training modification. Consequently, this study aimed to examine the segmental biomechanical variables associated with performance in the start phase of juvenile speed skaters from the Valle del Cauca Skating League, using exploratory variable-selection and cross-validation techniques to evaluate model stability and internal predictive validity.

## 2. Materials and Methods

### 2.1. Study Design

The study had a quantitative, observational, and analytical approach, with an explanatory cross-sectional design. Its aim was to examine cross-sectional associations between segmental biomechanical variables and performance in the start phase of the first 20 m in speed skaters, without modifying their usual training or performance conditions. Given the observational design, the study was not intended to establish causal relationships.

### 2.2. Sample and Participants

The sample consisted of 24 juvenile speed skaters aged 14–16 years, all affiliated with the Valle del Cauca Skating League. The participants were active competitive skaters engaged in systematic training at the time of data collection. Their training experience ranged from 6 to 8 years, and their usual training frequency was four sessions per week, with each session lasting approximately 90 to 120 min. Regarding competitive level, all athletes competed at the departmental level, and some had experience in national-level competitions. Therefore, the sample should be interpreted as trained youth skaters rather than recreational participants.

Regarding sex distribution, 16 athletes were female (code = 0), and 8 were male (code = 1). Body weight was also recorded because of its potential influence on acceleration capacity and segmental inertial estimates. These variables were examined descriptively to contextualize the sample and to identify possible sources of interindividual variability. However, because of the limited sample size and the primary objective of examining segmental biomechanical variables associated with performance, sex and body weight were not included as covariates in the final predictive model. Their potential influence was therefore considered when interpreting the results.

The inclusion criteria were as follows: being between 14 and 16 years of age, belonging to the Valle del Cauca Skating League, having systematic training experience in speed skating, participating regularly in training sessions, being actively involved in competitive skating, being free of musculoskeletal injury at the time of testing, and having both written informed consent from the legal guardian and assent from the athlete. The exclusion criteria were the presence of pain or musculoskeletal injury during the test, inability to complete the three 20 m attempts, irregular participation in training during the data collection period, or absence of informed consent or assent.

Biological maturation status was not directly assessed in this study. This is relevant because maturational differences may influence body mass, strength development, coordination, acceleration capacity, and segmental movement patterns in adolescent athletes. Therefore, the absence of maturation assessment was considered when interpreting the findings.

Prior to data collection, the skaters and their guardians were informed about the objectives and procedures of the study. Assent and informed consent were obtained in accordance with the ethical principles governing research involving human subjects. Participants performed the test using their usual competition equipment (helmet and skates), which ensured that the data obtained reflected ecologically valid conditions and were representative of their actual performance in the start phase.

### 2.3. Procedure

The research was conducted in an organized and standardized environment, promoting uniform conditions during the test. Tests were conducted on an adapted official track at the same time (4:00 p.m.).

Before the test, athletes completed a warm-up divided into two phases: a general phase that lasted 7 min and a 5 min test-specific phase. Participants gave their assent, and their guardians gave their consent for study participation.

### 2.4. Instruments

Data were extracted using Tracker (version 6.3.2), a free video-analysis software program developed by Open Source Physics. Each athlete was assigned an identification number to preserve confidentiality during the test. Subsequently, anatomical landmarks were marked using nonreflective markers. The study was conducted in accordance with the guidelines of the Ministry of Sport.

To minimize the measurement error inherent to two-dimensional video analysis, recordings were performed with the camera placed on a tripod. This helped ensure that the distance from the plane of motion and the camera framing remained constant across all attempts. Videos were acquired at a sampling rate of 60 frames per second (fps) and spatially calibrated in Tracker using a 1.00 m rigid segment arranged in the same plane as the skaters’ displacement. Although higher sampling frequencies, such as 120–240 fps, are preferable for the detailed analysis of rapid biomechanical events, the 60 fps acquisition used in this study was considered acceptable for the specific purpose of estimating global segmental kinematic patterns across the complete 20 m start phase. At 60 fps, each frame represents approximately 16.7 ms; therefore, considering that the 20 m times ranged from 3.12 to 3.93 s, each trial provided approximately 187–236 frames for analysis. This temporal resolution was considered sufficient to describe gross sagittal-plane segmental behavior during the start phase, but not to capture very short-duration events such as wheel-ground contact, impact peaks, or instantaneous joint accelerations. To reduce noise in derivative estimation (velocity and acceleration), the trajectories of the markers were smoothed using a second-order Savitzky–Golay filter with a five-frame window prior to the calculation of linear velocities, accelerations, and angular variables. This procedure helped stabilize kinematic signals, particularly accelerations, which are highly sensitive to noise when obtained by numerical differentiation of positions.

Videos were recorded using an iPhone 14 Pro smartphone (Apple Inc., Cupertino, CA, USA) at a sampling frequency of 60 frames per second. The device was mounted on a tripod and positioned laterally to the lane, perpendicular to the sagittal plane of displacement, maintaining a fixed field of view with no zoom variation.

The camera height was set close to the athletes’ center of mass, and the distance was sufficient to capture the entire analyzed phase. This arrangement was intended to minimize, but not eliminate, parallax effects. Because a single two-dimensional lateral view was used, the analysis was restricted to sagittal-plane motion; therefore, mediolateral displacements, transverse rotations, and out-of-plane segment movements could not be quantified and may have introduced tracking error. Spatial calibration was performed using the 1.00 m rigid reference segment positioned in the same plane as the skaters’ displacement; consequently, the scale was considered valid for the calibrated plane, whereas deviations outside that plane could introduce measurement error. For this reason, the results were interpreted as two-dimensional estimates of global segmental kinematics rather than complete three-dimensional biomechanical measurements. Performance was summarized as the shortest time recorded over the three attempts, with a standardized recovery period of 10 min between repetitions. The best attempt was retained because the objective was to characterize each athlete’s maximal start performance under ecologically valid competitive conditions, where peak execution capacity is commonly prioritized. All participants completed the same number of attempts and had the same recovery period, which helped standardize the opportunity to achieve maximal performance. Nevertheless, this outcome should be interpreted as best-trial performance rather than average performance across attempts.

The anatomical points used for the analysis of the upper limb were the lateral epicondyle, the styloid process of the ulna, and the acromion. For the lower limb, they were the greater trochanter and the lateral femoral epicondyle.

For body-segment analysis, the center of mass was determined from two anatomical points. In other words, the body segments were defined as follows: the arm segment consisted of the lateral epicondyle and the acromion; the forearm segment consisted of the styloid process of the ulna and the lateral epicondyle; the torso consisted of the acromion and the greater trochanter; and the thigh consisted of the lateral epicondyle of the femur and the greater trochanter.

Body-segment kinematic variables were calculated using Zatsiorsky’s cadaver-based method, as adjusted by De Leva [21] in which the center of mass corresponded to a percentage of the total mass of the individual, with variations between men and women. The segmental inertial force estimate was calculated by multiplying the estimated mass of each body segment by its linear acceleration. The segmental inertial power estimate was then calculated as the product of the segmental inertial force estimate and the corresponding segmental velocity, using the following expression: segmental inertial power estimate = segmental inertial force estimate × segmental velocity.

In this context, the terms “force” and “power” do not refer to directly measured external mechanical variables, such as ground reaction forces or power obtained from force platforms. Instead, they represent segmental inertial estimates derived from two-dimensional kinematics and De Leva’s anthropometric parameters. Therefore, throughout the manuscript, these variables are interpreted as kinematic proxies of segmental inertial behavior and not as direct measurements of force or mechanical power.

Photoelectric cells were used to record the best time achieved over the first 20 m of the start phase, in seconds and milliseconds. Three attempts were performed, during which participants used their helmets and competition skates.

To reduce digitizing variability, anatomical marking and video digitization were performed by a single trained evaluator following a standardized protocol and applying the same marker-placement and tracking criteria across all videos. Trajectory continuity and the absence of spurious jumps were visually checked before calculating the derivatives, and corrective redigitization was performed when obvious discontinuities were identified. These procedures constituted quality-control measures intended to reduce evident tracking errors; however, they should not be interpreted as evidence of measurement reliability. Corrective redigitizations were not conducted as independent, blinded test–retest measurements. Because no prespecified repeated-digitization protocol and no independent second evaluator were available, intra-evaluator reliability, inter-evaluator reliability, and absolute measurement-error indices such as the intraclass correlation coefficient, coefficient of variation, standard error of measurement, and typical error could not be estimated.

### 2.5. Statistical Analysis

Data were analyzed in Python (version 3.12) using specialized statistical and machine learning libraries (pandas, numpy, scikit-learn, statsmodels, PyMC, and ArviZ). The procedure was structured in several complementary stages to identify the most relevant biomechanical predictors of time in the first 20 m of juvenile skaters and to evaluate the stability and predictive capacity of the models obtained.

The analytical strategy was organized as a hierarchical exploratory framework rather than as a set of independent confirmatory models. LASSO with LOOCV was used to reduce a relatively large, correlated, and partially redundant feature set and to identify candidate predictors. The post-selection OLS model was fitted to express the selected associations in the original measurement units and to provide conventional regression diagnostics. Bootstrap resampling was used to examine the sensitivity of the estimated coefficients to sample fluctuations, whereas Bayesian regression was used to characterize posterior direction and uncertainty under weakly informative priors. Prior-sensitivity analysis and posterior predictive checks were treated as diagnostics of prior dependence and model–data agreement, respectively, rather than as additional sources of independent confirmation. Nested LOOCV was considered the primary assessment of internal predictive performance because preprocessing, tuning, variable selection, and model fitting were repeated within each training fold. None of these procedures was considered capable of compensating for the small sample size or replacing replication and external validation.

First, basic descriptive statistics (mean, standard deviation, minimum, and maximum values) were calculated for all variables of interest to characterize the distribution of the data and verify the consistency of the recorded values. Subsequently, linear associations between each predictor and the response variable (20 m time) were explored using Pearson correlation coefficients. Because Pearson correlation assumes linear relationships, scatterplots were visually inspected to assess the plausibility of linear patterns between the main candidate variables and 20 m time. These bivariate correlations were interpreted as descriptive and exploratory screening indicators, not as confirmatory hypothesis tests. Given the small sample size, the possibility of nonlinear associations could not be completely ruled out.

Because multiple bivariate correlations were examined before LASSO selection, no formal multiple-comparison correction was applied to the bivariate correlation matrix. These correlations were used as exploratory screening measures and to describe the structure of the candidate feature set, not as confirmatory hypothesis tests or as the basis for final inferential conclusions.

Sex and body weight were treated as contextual variables because both may influence acceleration capacity and segmental inertial estimates in adolescent skaters. Sex was coded as 0 = female and 1 = male, and body weight was recorded in kilograms. These variables were examined descriptively and used to contextualize interindividual variability in performance. Body weight was also explored in the bivariate correlation analysis. However, sex and body weight were not included as primary covariates in the final predictive model because of the limited sample size, the imbalance between female and male athletes, and the fact that body weight was already indirectly incorporated into the segmental inertial estimates derived from the anthropometric model. To evaluate their possible influence, an exploratory sensitivity analysis was performed by adding sex and body weight to the final biomechanical model.

For multivariate selection, penalized Least Absolute Shrinkage and Selection Operator (LASSO) regression with leave-one-out cross-validation (LOOCV) was used. This procedure allowed the retention of only the variables with the greatest contribution to the model, reducing model complexity and favoring parsimony. However, given the small sample size (*n* = 24) and the number of candidate biomechanical predictors, LASSO-based variable selection was considered exploratory and potentially sensitive to sample fluctuations. Therefore, the selected predictors should not be interpreted as a definitive set of biomechanical determinants but as the most consistent candidates identified within this dataset. Predictors were limited to a maximum of five, following stability criteria given the available sample size.

It should also be noted that several candidate predictors were mathematically related because they were derived from common segmental kinematic signals. For example, the segmental inertial force estimate was calculated from segmental mass and acceleration, whereas the segmental inertial power estimate was calculated from the segmental inertial force estimate and segmental velocity. Therefore, acceleration, segmental inertial force estimates, velocity, and segmental inertial power estimates should not be interpreted as fully independent biomechanical constructs. In this context, LASSO was used as an exploratory variable-reduction procedure to identify representative predictors among correlated and partially redundant variables, rather than as a method for establishing independent causal determinants.

Subsequently, an ordinary least squares (OLS) linear regression model was fitted, incorporating the selected predictors and estimating the coefficients by least squares. To strengthen the inference, standard errors corrected for heteroskedasticity (HC3) were calculated. Multicollinearity was evaluated using the Variance Inflation Factor (VIF), considering critical values of 5 and 10 as reference points.

Influential observations were evaluated using Cook’s distance. Given the small sample size, the conventional threshold of 4/n was used as a screening criterion to identify observations with potentially significant influence on the regression coefficients. Because this analysis was exploratory, Cook’s distance was interpreted as a diagnostic tool rather than as a criterion for automatic exclusion.

Because LASSO-based predictor selection uses information contained in the data, the significance tests of the subsequent OLS model may be affected by selection bias. Consequently, the post-selection *p*-values were interpreted as descriptive and exploratory. Nested LOOCV was treated as the principal assessment of internal predictive performance, whereas bootstrap resampling and Bayesian modeling were used as secondary assessments of coefficient sensitivity and uncertainty. Agreement across these analyses was interpreted as within-sample sensitivity evidence and not as independent replication.

To evaluate predictive ability while minimizing data leakage, a nested LOOCV procedure was implemented. In each outer LOOCV iteration, one participant was held out as the test case. All preprocessing steps, including standardization, were estimated only from the remaining training data. Within each training set, LASSO tuning and variable selection were repeated, and only the variables selected within that training fold were used to fit the regression model. The fitted model was then applied to the held-out participant, who had not contributed to scaling, LASSO tuning, variable selection, or model fitting. This process was repeated until each participant had served once as the test case. Predictive performance was summarized using the root mean square error (RMSE), mean absolute error (MAE), and predictive coefficient of determination (R^2^_pred). The final model reported in the tables was fitted using the full dataset only for interpretative purposes and should not be confused with the fold-specific models used for nested predictive validation.

Finally, Bayesian linear regression was implemented in Python version 3.11.9 using PyMC version 5.16.2. Posterior diagnostics, posterior predictive checks, and Pareto-smoothed importance-sampling leave-one-out cross-validation (PSIS-LOO) were performed using ArviZ version 0.18.0. Prior to model fitting, both the outcome variable (20-m start time) and the predictors were standardized to a mean of zero and a standard deviation of one. Weakly informative Normal(0, 1) priors were assigned to the intercept and regression coefficients, and a HalfNormal(1) prior was assigned to the residual standard deviation. This standardization placed the model parameters on comparable scales and facilitated the interpretation of the prior distributions. For practical interpretation and the calculation of predictive performance metrics, posterior estimates and predictions were transformed back to the original outcome scale in seconds.

Bayesian sampling was performed using four chains with 5000 iterations per chain. Model convergence was evaluated using multiple diagnostic criteria, including visual inspection of trace plots, the potential scale reduction factor (R_hat), and effective sample size indicators (ESS_bulk and ESS_tail). R_hat values close to 1.00 and adequate effective sample sizes were considered evidence of stable posterior sampling. Posterior parameter distributions, 94% highest density intervals (HDIs), and posterior sign probabilities were calculated for each selected variable. In addition, a prior sensitivity analysis was performed by comparing the main weakly informative Normal(0,1) prior specification with alternative Normal priors of different scales, Normal(0,0.5) and Normal(0,2), to evaluate whether posterior estimates were robust to reasonable changes in prior assumptions.

Bayesian posterior predictive performance was quantified using complementary metrics in the original outcome units. Specifically, Bayesian R^2^, posterior predictive RMSE, posterior predictive MAE, the empirical coverage of the 94% posterior predictive intervals, and the mean width of these intervals were calculated. These metrics were interpreted as internal posterior predictive fit indices and not as evidence of external predictive validity.

## 3. Results

Data from 24 juvenile skaters were analyzed. The time recorded in the start phase of the first 20 m ranged between 3.12 s and 3.93 s, with a mean of 3.48 s and a standard deviation of 0.17 s. Body weight ranged from 47.95 kg to 81.00 kg (M = 56.76; SD = 8.33). Regarding sex, there were 16 female athletes (code = 0) and 8 male athletes (code = 1). When the 20 m time was stratified according to this coding, the female group had a mean time of 3.53 s (SD = 0.18), and the male group had a mean time of 3.38 s (SD = 0.12). Regarding body weight by subgroup, code 0 showed a mean of 55.79 kg (SD = 7.56), and code 1 showed a mean of 58.69 kg (SD = 9.95) (see Table 1).

Sex and body weight were interpreted as contextual variables rather than as primary biomechanical predictors. In the bivariate analysis, body weight showed a moderate negative association with 20 m time, suggesting that heavier athletes tended to record shorter times. Likewise, male athletes showed lower mean 20 m times than female athletes. However, these patterns were interpreted cautiously because of the small sample size and the unequal distribution by sex. In the exploratory sensitivity model that added sex and body weight to the selected biomechanical predictors, sex showed a negligible adjusted coefficient, whereas body weight showed a negative but uncertain association with 20 m time. The direction of the main biomechanical predictors remained generally stable, although the effect of some segmental variables was attenuated. Therefore, the final model retained the biomechanical predictors selected by LASSO, while sex and body weight were considered contextual variables for interpretation.

The biomechanical variables obtained using Tracker software and estimated using the body-segment method showed interindividual variability in velocity, acceleration, segmental inertial force estimates, and segmental inertial power estimates across the different body segments.

The Shapiro–Wilk analysis suggested deviations from normality for several selected predictors: forearm segmental inertial force estimate (W = 0.822, *p* = 0.001), thigh velocity (W = 0.913, *p* = 0.040), forearm velocity (W = 0.912, *p* = 0.039), and thigh angular velocity (W = 0.860, *p* = 0.003). Torso angular acceleration did not show evidence of departure from normality (W = 0.942, *p* = 0.177). These results support the exploratory interpretation of the bivariate analyses and reinforce the need for cautious interpretation in a small sample.

**Figure 1:**
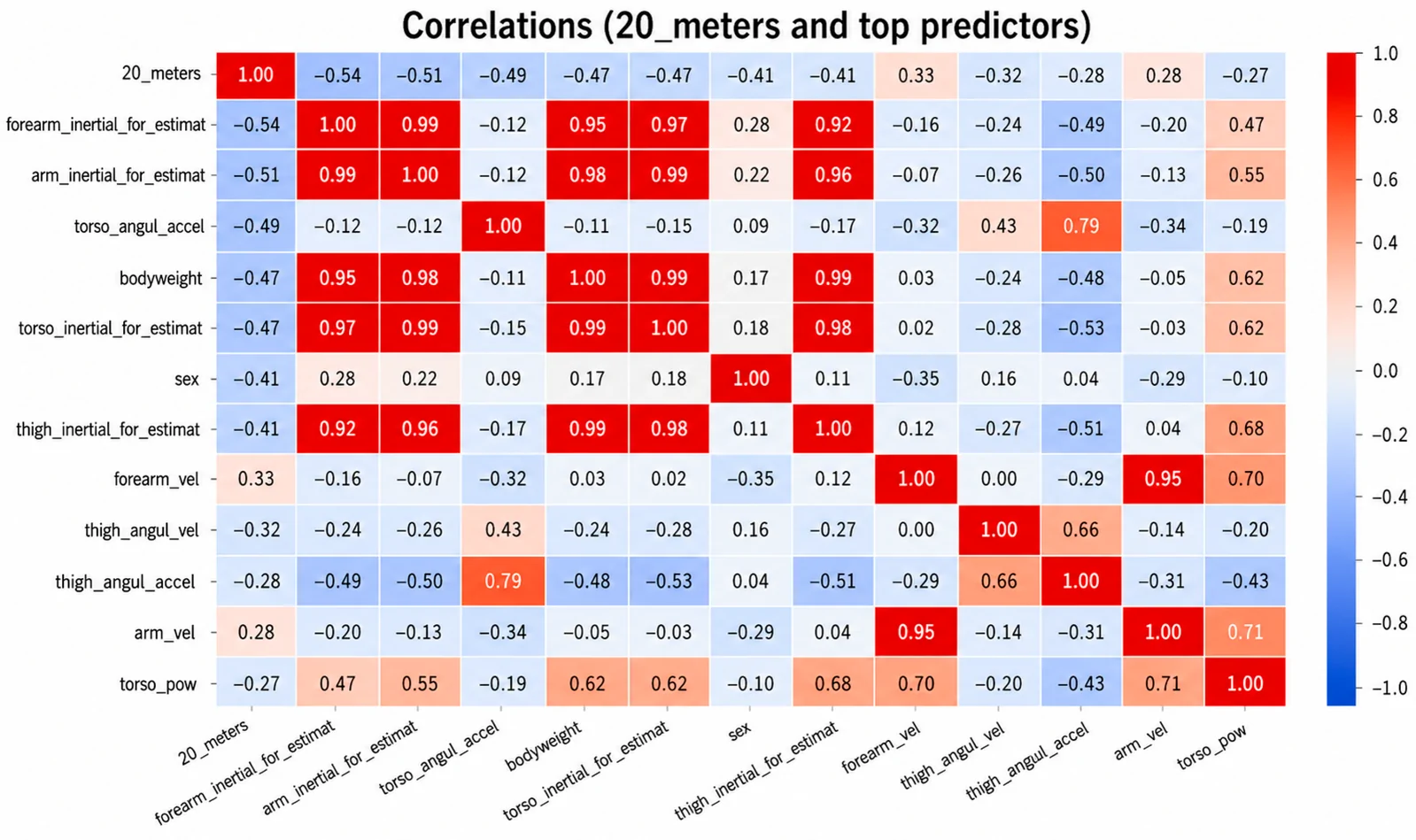
Pearson bivariate correlations among performance and predictor variables. Note: forearm_inertial_for_estimat = forearm segmental inertial force estimate; arm_inertial_for_estimat = arm segmental inertial force estimate; torso_angul_accel = torso angular acceleration; torso_inertial_for_estimat = torso segmental inertial force estimate; thigh_inertial_for_estimat = thigh segmental inertial force estimate; forearm_vel = forearm velocity; thigh_angul_vel = thigh angular velocity; thigh_angular_accel = thigh angular acceleration; arm_vel = arm velocity; torso_pow = torso segmental inertial power estimate.

The bivariate correlation analysis indicated that the predictors most strongly associated with performance (20 m time) were the forearm segmental inertial force estimate (r = −0.54), arm segmental inertial force estimate (r = −0.51), and torso angular acceleration (r = −0.49), followed by body weight (r = −0.47) and torso segmental inertial force estimate (r = −0.47). Conversely, forearm and arm velocity were positively correlated with time (r = 0.33 and r = 0.28, respectively) (see Figure 1).

The correlation matrix also showed very high pairwise correlations among several candidate variables, with some coefficients exceeding r = 0.95. This pattern indicates substantial redundancy within the initial biomechanical feature set, particularly among variables derived from shared kinematic and anthropometric components. Therefore, the correlation matrix should not be interpreted as evidence that the candidate variables represented independent biomechanical constructs. Instead, it supports the need to interpret the subsequent LASSO procedure as an exploratory variable-reduction strategy applied to a correlated and partially redundant set of derived features.

Variable selection using LASSO with LOOCV identified the forearm segmental inertial force estimate, thigh velocity, forearm velocity, thigh angular velocity, and torso angular acceleration as the most consistent candidate variables within this dataset. These variables were retained because they provided a parsimonious representation of the correlated biomechanical feature set after penalized selection and collinearity assessment in the final model. Nevertheless, their simultaneous inclusion should not be interpreted as evidence that they are biomechanically independent constructs; rather, they should be understood as representative variables selected from a mathematically related and partially redundant set of features.

In the post-selection heteroskedasticity-robust OLS model (HC3), the estimated coefficients were β = −1.01 for the forearm segmental inertial force estimate, β = −1.94 for thigh velocity, β = 1.44 for forearm velocity, β = −0.034 for thigh angular velocity, and β = −0.030 for torso angular acceleration. These coefficients should be interpreted as exploratory post-selection associations with 20 m time. Because the variables were selected through a data-driven LASSO procedure in a small cross-sectional sample, the *p*-values reported in Table 2 should be considered descriptive post-selection statistics rather than conventional confirmatory inferential tests. The multicollinearity analysis showed that VIF values were below 5 for most variables, except thigh velocity, which showed a value slightly above this threshold. However, although all VIF values were below 10 in the final model, this does not eliminate the substantial bivariate redundancy observed among several candidate variables in the initial feature set.

Influence diagnostics showed that three observations had Cook’s distance values above the conventional 4/n threshold (4/24 = 0.167), with a maximum Cook’s distance of 1.550. A sensitivity analysis excluding these influential observations preserved the direction of the main coefficients, although some coefficient magnitudes changed. Therefore, the regression results should be interpreted cautiously, as expected in a small sample where individual observations may have a substantial impact on model estimates.

Nested LOOCV showed an RMSE of 0.12 s and a MAE of 0.08 s, with a predictive coefficient of determination (R^2^_pred) of 0.485. This suggests that the model accounted for 49% of the variability in start time under nested internal cross-validation conditions, with small errors relative to the observed range (3.1–3.9 s).

**Figure 2:**
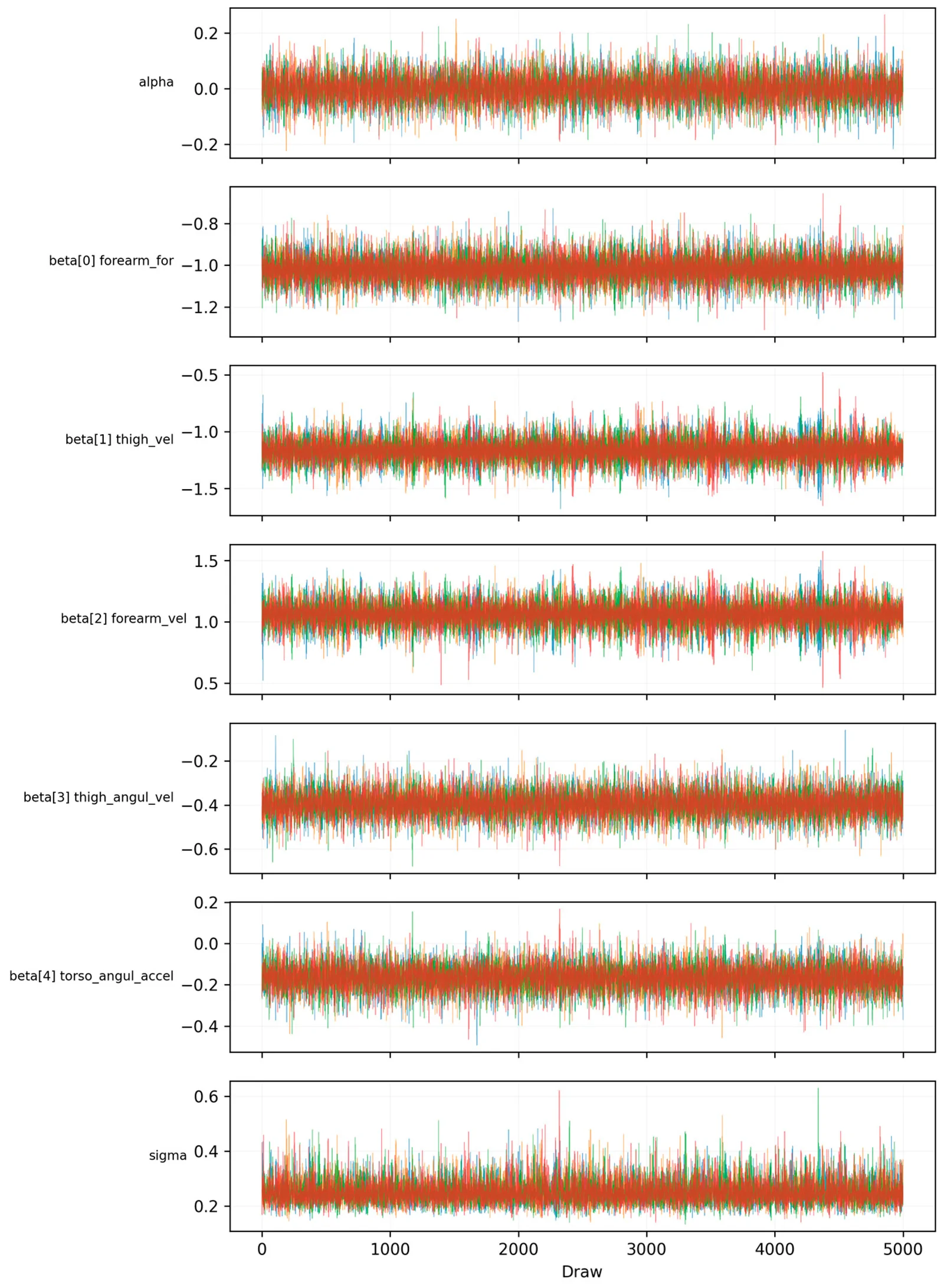
Trace plots for the Bayesian linear regression model parameters. Note. Each color represents one of the four independently initialized Markov chain Monte Carlo (MCMC) chains. The horizontal axis represents posterior draws, and the vertical axis shows the sampled values of each model parameter. The substantial overlap among chains, stable central tendency, and absence of persistent trends or chain-specific separation provide visual evidence of adequate mixing and stationarity. Visual inspection of the trace plots was complemented by the $\hat{R}$, bulk effective sample size, and tail effective sample size diagnostics reported in Table 4.

Bootstrap resampling (B = 2000) supported the stability of the coefficients for the forearm segmental inertial force estimate, thigh velocity, and forearm velocity, whose empirical intervals were clearly separated from zero. Thigh angular velocity showed a small but consistent negative association. In contrast, torso angular acceleration showed greater uncertainty because its bootstrap interval included values close to or above zero, indicating that this association was less stable and should be interpreted with caution (see Table 3) Before interpreting the Bayesian parameter estimates, visual inspection of the Markov chain trace plots indicated adequate chain mixing and no apparent evidence of non-stationarity across the four chains (Figure 2).

**Table 3:** Bootstrap estimates of the coefficients in the regression model for predicting 20 m times using selected biomechanical variables.

Parameter	Mean_Boot	p2_5	p50	p97_5
const	4.7	4.1	4.7	5.0
forearm_inertial_for_estimat	−0.98	−1.2	−1.00	−0.60
thigh_vel	−2.0	−2.6	−2.0	−1.5
forearm_vel	1.5	1.1	1.5	2.0
thigh_angul_vel	−0.034	−0.049	−0.034	−0.023
torso_angul_accel	−0.024	−0.045	−0.027	0.017

Note: mean_boot = bootstrap mean; p2_5 = 2.5th percentile; p50 = 50th percentile or bootstrap median; p97_5 = 97.5th percentile. The coefficients correspond to the variables included in the robust model: forearm_inertial_for_estimat = forearm segmental inertial force estimate; thigh_vel = thigh velocity; forearm_vel = forearm velocity; thigh_angul_vel = thigh angular velocity; torso_angul_accel = torso angular acceleration.

**Table 4:** Posterior parameters and convergence diagnostics of the Bayesian linear regression model for predicting 20 m start time.

Parameter	Mean	SD	HDI_3%	HDI_97%	R_Hat	ESS_Bulk	ESS_Tail
alpha	−0.001	0.052	−0.097	0.099	1.00	23,630	12,875
beta[0]	−1.019	0.066	−1.144	−0.894	1.00	43,326	12,769
beta[1]	−1.169	0.115	−1.389	−0.956	1.00	86,020	6623
beta[2]	1.060	0.111	0.845	1.266	1.00	86,020	7851
beta[3]	−0.395	0.060	−0.508	−0.281	1.00	18,668	14,469
beta[4]	−0.166	0.067	−0.293	−0.043	1.00	20,579	13,728
sigma	0.252	0.046	0.174	0.339	1.00	36,822	10,695

Note: SD = standard deviation; HDI_3% = lower bound of the 94% highest density interval; HDI_97% = upper bound of the 94% highest density interval; R_hat = potential scale reduction factor; ESS_bulk = bulk effective sample size; ESS_tail = tail effective sample size. The coefficients correspond to the standardized variables included in the Bayesian model: beta[0] = forearm segmental inertial force estimate; beta[1] = thigh velocity; beta[2] = forearm velocity; beta[3] = thigh angular velocity; beta[4] = torso angular acceleration. R_hat values close to 1.00 and adequate ESS values were interpreted as evidence of satisfactory convergence and posterior sampling stability.

**Figure 3:**
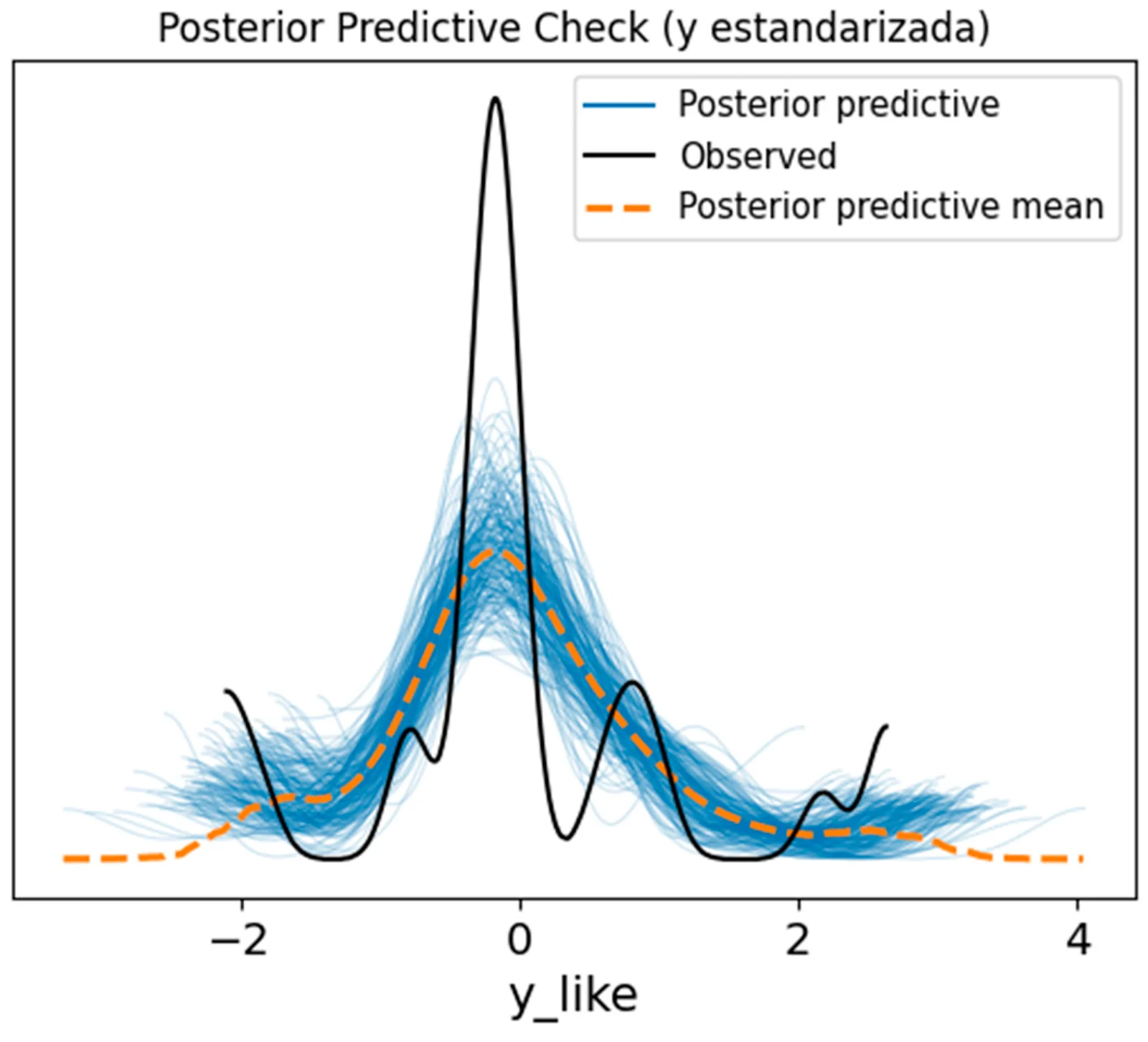
Predictive validation of the Bayesian model using posterior predictive checks and Bayesian leave-one-out cross-validation. Note. Posterior predictive check of the Bayesian model for the standardized outcome variable. The observed distribution is shown in black, while the light blue curves represent replicated posterior predictive distributions, and the dashed orange curve represents the posterior predictive mean. The model captures the general distributional pattern of the data, although some discrepancies are observed around the central peak and local density fluctuations.

Posterior predictive checks showed that the model reproduced the general empirical distribution of 20 m times, although some local discrepancies were observed around the central density peak. Quantitatively, the Bayesian model showed a Bayesian R^2^ of 0.938 (94% HDI: 0.882–0.968). The RMSE of the posterior mean predictions was 0.035 s, and the MAE was 0.026 s. When residual uncertainty was incorporated through posterior predictive replicated outcomes, the posterior predictive RMSE was 0.059 s (94% HDI: 0.042–0.082), and the posterior predictive MAE was 0.047 s (94% HDI: 0.033–0.067). The empirical coverage of the 94% posterior predictive intervals was 100%, with a mean interval width of 0.184 s. Bayesian LOO validation reported an elpd_loo of −4.60 (SE = 3.88) and an effective complexity (*p*_loo) of 6.73, with good_k = 0.7, indicating moderate stability in this small sample (see Figure 3). These metrics should be interpreted as internal posterior predictive fit indices rather than as evidence of external predictive validity.

The Bayesian approach was directionally consistent with the associations observed in the post-selection OLS model (Table 4). Convergence diagnostics supported the adequacy of posterior sampling: R_hat values were 1.00 for all parameters, and ESS_bulk and ESS_tail values were adequate, indicating stable estimation of posterior distributions. Visual inspection of the trace plots showed satisfactory mixing across chains and no visible evidence of non-stationarity. Posterior distributions suggested non-zero associations for the forearm segmental inertial force estimate (β = −1.019; 94% HDI −1.144 to −0.894), thigh velocity (β = −1.169; 94% HDI −1.389 to −0.956), forearm velocity (β = 1.060; 94% HDI 0.845 to 1.266), thigh angular velocity (β = −0.395; 94% HDI −0.508 to −0.281), and torso angular acceleration (β = −0.166; 94% HDI −0.293 to −0.043). However, given the small sample size, the data-driven variable-selection process, and the bootstrap uncertainty observed for torso angular acceleration, these Bayesian estimates should be interpreted as complementary evidence regarding direction and uncertainty, not as confirmatory evidence of independent biomechanical effects.

Prior sensitivity analysis showed that the direction of the posterior coefficients remained stable when alternative Normal priors with different scales were used. Under Normal(0,0.5), Normal(0,1), and Normal(0,2) priors, the forearm segmental inertial force estimate, thigh velocity, and thigh angular velocity consistently showed negative associations with 20 m start time, whereas forearm velocity consistently showed a positive association. The estimate for torso angular acceleration also remained negative across prior specifications, although its interpretation should remain cautious because of the uncertainty observed in the bootstrap analysis. Overall, the sensitivity analysis suggested that the main directional conclusions were not driven exclusively by the chosen weakly informative prior specification.

Finally, interpretation in the original units showed that a one-SD increase in thigh velocity and in the forearm segmental inertial force estimate was associated with decreases of 0.20 s and 0.17 s, respectively, in 20 m time. In contrast, a one-SD increase in forearm velocity predicted a +0.18 s increase. Thigh angular velocity and torso angular acceleration showed more modest effects, corresponding to decreases of 0.07 s and 0.03 s, respectively.

## 4. Discussion

This study examined segmental biomechanical variables associated with performance in the 20 m start phase using Tracker software. The exploratory regression model indicated that the forearm segmental inertial force estimate, thigh velocity, and thigh angular velocity were associated with shorter start times, whereas forearm velocity was associated with longer start times. Torso angular acceleration also showed a negative association with start time, although this finding was less stable in the bootstrap analysis and should therefore be interpreted with greater caution. Because of the observational cross-sectional design, these findings should be interpreted as statistical associations and not as evidence that these variables causally determine sprint performance.

The literature integrating video analysis and biomechanical variables in skaters remains scarce. To the best of our knowledge, only one study identified in our search strategy, conducted by Knox and Comstock [24], focused on the impact of falls in ice skaters. This suggests that the proposed methodological approach has been scarcely explored in speed skating research. However, the reliability of the video analysis should be further examined to determine whether the derived kinematic variables can be extrapolated to other contexts and populations. Along these lines, Dingenen et al. [25] evaluated kinematic variables by video analysis and reported intraclass correlation coefficients (ICCs) between 0.90 and 0.99, indicating the high reliability of this technique. Similarly, Pipkin et al. analyzed running technique in the sagittal and frontal planes and found that most of the variables had moderate to very good reliability levels (0.60–0.90).

When contrasted with previous research, the present findings are consistent with evidence suggesting that knee flexion, hip abduction, and external rotation are relevant movement features during skating [8,13]. In addition, previous studies have reported associations between peak velocity and stride length [8]. Accordingly, our findings suggest that linear velocity and thigh angular velocity may be relevant variables to monitor during the start phase, although their role should be interpreted as associative rather than causal.

In contrast, the positive association between forearm velocity and 20 m time may be compatible with a less coordinated arm-swing pattern in this sample. However, movement coordination was not quantified directly, as the study did not assess intersegmental phase relationships, arm–leg coupling, temporal synchronization, or coordinative variability. Therefore, this coordination-based explanation remains speculative and should be regarded as a biomechanically plausible hypothesis rather than as a mechanism demonstrated by the present data. The observed association only indicates that greater forearm velocity co-occurred with longer start times in this sample; it does not establish that faster forearm motion caused poorer coordination or reduced performance. Upper-limb movement should therefore be interpreted in relation to whole-body movement organization rather than as an isolated determinant of performance. Similarly, the forearm and arm segmental inertial force estimates may reflect a modulating role of upper-limb motion. Nevertheless, these estimates should not be interpreted as direct measurements of upper-limb force or as causal contributors to propulsion. Rather, they may represent kinematic indicators associated with whole-body displacement during the start phase [26,27].

Torso angular acceleration also showed a negative association with start-phase time in the post-selection regression and Bayesian models. However, this variable should be interpreted with greater caution than the other selected variables because its bootstrap interval included values close to or above zero. Therefore, the finding should be considered tentative. It may suggest that anterior trunk motion is related to the orientation of segmental movement during the start phase, but it cannot be concluded that torso angular acceleration is a stable or causal determinant of performance. Future longitudinal or experimental studies are needed to clarify whether trunk angular acceleration is a robust biomechanical marker or simply a sample-specific association.

The multistage analytical strategy was intended as a hierarchical sensitivity framework rather than as a collection of independent confirmatory analyses. LASSO reduced the correlated candidate feature set, the post-selection OLS model provided interpretable coefficients in the original units, bootstrap resampling assessed sensitivity to sample fluctuations, and nested LOOCV evaluated the internal predictive performance of the complete selection pipeline. Bayesian regression was used to characterize posterior uncertainty, while prior-sensitivity analysis and posterior predictive checks evaluated dependence on the prior specification and model–data agreement, respectively. Importantly, all of these procedures analyzed the same 24 participants; therefore, their agreement does not constitute independent replication, increase the effective sample size, or eliminate the risk of overfitting. The Bayesian results should consequently be interpreted as complementary uncertainty analyses within an exploratory framework and not as confirmatory evidence.

Taken together, the predictors retained by LASSO should be treated as candidate biomechanical variables identified within this specific sample rather than as stable determinants of 20 m start performance. The reproducibility of the selected variable set, the direction and magnitude of the estimated associations, and their predictive contribution need to be examined in larger independent cohorts and through external validation before these findings can be considered robust or generalizable. Future replication studies should also determine whether the observed associations remain consistent across sex, biological maturation status, competitive level, and measurement settings.

Sex-related differences also warrant a more cautious interpretation of the present findings. Although an exploratory sensitivity analysis suggested that adding sex to the final model did not substantially modify the direction of the selected biomechanical predictors, the limited sample size and the unequal distribution of female (*n* = 16) and male (*n* = 8) inline skaters precluded adequately powered sex-specific analyses or the evaluation of interaction effects. This limitation is particularly relevant because adolescence is characterized by substantial interindividual variability in biological maturation, which influences muscle mass, strength, neuromuscular coordination, sprint acceleration, and movement mechanics. Consequently, the shorter 20 m times observed in male athletes should not be interpreted as evidence that the same biomechanical determinants operate similarly across sexes, but rather as descriptive differences that require confirmation in larger cohorts. Future studies should incorporate biological maturation indicators (e.g., maturity offset or peak height velocity) and evaluate sex-stratified or interaction models to determine whether the biomechanical predictors identified in this exploratory analysis remain consistent after accounting for developmental status and sex-specific neuromuscular adaptations [28,29,30].

Because no independent validation cohort was available and the sample was small, age-restricted, and drawn from a single regional skating league, the transportability of the selected variables and coefficient estimates to other athletes, competitive levels, or measurement settings has not been established. No decision thresholds, reference values, or classification rules were developed or validated. Therefore, the present findings should not be used for athlete selection, talent identification, ranking, exclusion, or individualized training decisions. At this stage, their appropriate role is limited to provisional performance monitoring and hypothesis generation pending formal reliability assessment, independent replication, and external validation.

This study had several limitations. First, specific information on speed skating in the start phase of the first 20 m is scarce. Therefore, evidence from other types of skating, such as ice skating or figure skating, had to be considered to support the interpretation of the findings. Second, the absence of a formal measurement-reliability assessment constitutes a major methodological limitation of this study. Although marker placement and video digitization were performed by a single trained evaluator following a standardized protocol, and trajectories were visually inspected to identify obvious discontinuities, these quality-control procedures do not demonstrate either measurement repeatability or reproducibility. The corrective redigitizations conducted after detecting trajectory discontinuities were not independent test–retest measurements and therefore could not be used to estimate reliability. No prespecified repeated digitization was performed by the same evaluator, and no independent second evaluator was available. Consequently, intra-evaluator and inter-evaluator intraclass correlation coefficients, coefficients of variation, standard errors of measurement, typical errors of measurement, and digitization errors were not quantified. The extent to which measurement error contributed to the observed interindividual variability therefore remains unknown. This limitation is particularly important because velocity and acceleration were obtained through numerical differentiation of marker trajectories, and measurement error in these variables may propagate to the derived segmental inertial force and power estimates. Accordingly, the precision and reproducibility of the reported biomechanical estimates cannot be established from the present data, and the findings should be interpreted with additional caution. Future studies should incorporate independent repeated digitizations by the same evaluator and by additional evaluators, report both relative and absolute reliability indices, and quantify measurement error before these variables are used in confirmatory, predictive, or explanatory models.

A further limitation concerns the observational cross-sectional design. Although the statistical models identified variables associated with 20 m start time, the study design does not allow for causal inference. Therefore, the observed associations should not be interpreted as evidence that modifying forearm segmental inertial force estimates, thigh velocity, thigh angular velocity, forearm velocity, or torso angular acceleration would necessarily change sprint performance. Longitudinal and experimental studies are required to determine whether these variables are modifiable causal factors or simply markers associated with performance level.

Another important limitation concerns the mathematical dependence among several biomechanical predictors. Some variables were not measured as independent mechanical constructs but were derived from shared components. Specifically, segmental inertial force estimates were calculated from segmental mass and acceleration, whereas segmental inertial power estimates were calculated from segmental inertial force estimates and segmental velocity. Consequently, acceleration, velocity, segmental inertial force estimates, and segmental inertial power estimates are partly interdependent. This mathematical structure may introduce redundancy among candidate predictors and may influence the variable-selection process. Therefore, LASSO should be understood as selecting representative variables from a set of correlated and partially dependent biomechanical features, rather than identifying independent biomechanical mechanisms. Future studies should consider dimension-reduction strategies, hierarchical feature selection, or pre-specified biomechanical domains to reduce redundancy among derived variables.

A further statistical limitation concerns the small sample size relative to the number of biomechanical variables initially extracted. Although LASSO regularization, bootstrap resampling, Bayesian estimation, and nested LOOCV were used to reduce overfitting and evaluate internal predictive performance, these procedures do not completely eliminate the risk of model optimism in a sample of only 24 participants. Variable selection is inherently data-driven and may be unstable when many candidate predictors are evaluated in a small dataset. Therefore, the predictors identified in this study should be interpreted as exploratory candidate predictors rather than definitive biomechanical determinants of 20 m start performance. Future studies with larger samples and external validation cohorts are needed to confirm the stability, generalizability, and practical relevance of these associations.

In addition, multiple bivariate correlations were examined before the LASSO selection stage. These correlations were used descriptively to explore the structure of the candidate feature set and to guide interpretation, not as confirmatory tests of independent hypotheses. Therefore, no formal multiple-comparison correction was applied. This should be considered when interpreting the correlation matrix, particularly given the small sample size and the redundancy among derived biomechanical variables.

Another methodological limitation concerns the definition of performance as the best of three attempts. This decision was consistent with the aim of capturing maximal start performance; however, it may yield slightly faster values than the mean of the three trials and may therefore introduce a positive performance bias. Consequently, the outcome should be interpreted as best-trial start performance rather than average start performance. Future studies should report both the best attempt and the mean or variability across attempts to provide a more complete profile of performance consistency.

An additional consideration when interpreting the present findings is the potential influence of biological maturation. Although participants were restricted to a relatively narrow chronological age range (14–16 years), chronological age does not necessarily reflect biological maturity during adolescence. Athletes of the same chronological age may differ substantially in skeletal development, hormonal status, muscle mass, neuromuscular function, and sprint capacity depending on their maturation stage. Consequently, part of the variability attributed to the selected biomechanical variables may also reflect maturational differences that were not quantified in the present study. The inclusion of validated maturity indicators, such as maturity offset or years from peak height velocity (PHV), would allow future studies to distinguish developmental effects from genuine biomechanical determinants of start performance and improve the interpretation of movement strategies in adolescent speed skaters [28,29,31].

Another limitation concerns the use of two-dimensional video analysis, which imposes inherent methodological constraints. Although the camera was positioned laterally and perpendicular to the sagittal plane to reduce measurement error, the system could not capture movements occurring outside the calibrated plane, such as mediolateral displacement, transverse rotations, or segmental deviations toward or away from the camera. This is particularly relevant in speed skating because the movement is not exclusively sagittal; frontal-plane mechanics, including lateral push-off, mediolateral weight transfer, edge control, and side-to-side displacement, may contribute meaningfully to start performance but were not quantified in the present analysis. Therefore, possible out-of-plane motion and residual parallax error may have affected the precision of marker tracking and the estimation of derived kinematic variables. In addition, spatial calibration was performed using a rigid reference segment located in the plane of displacement; consequently, the calibration was valid for that plane but may not fully correct positional deviations outside it. These two-dimensional measurement constraints, together with the absence of formal reliability estimates described above, should be considered when interpreting the segmental kinematic and inertial estimates. Future studies should address these limitations using three-dimensional motion capture or multiple synchronized cameras, together with prespecified intra-evaluator and inter-evaluator reliability protocols.

The distribution of the selected predictors was explored using the Shapiro–Wilk test. These tests were used descriptively to characterize the variables and to identify possible deviations from normality. However, predictor normality was not considered a strict assumption for the OLS model; model diagnostics focused primarily on residual behavior, influential observations, and predictive stability.

In addition, although sex and body weight were examined descriptively and in an exploratory sensitivity analysis, the sample size and the unequal distribution between female and male athletes did not allow for robust sex-stratified models or fully adjusted multivariable models. Moreover, body weight was conceptually related to the segmental inertial estimates because these variables were derived using anthropometric parameters. Therefore, the final model should be interpreted as a biomechanical predictive model rather than as a fully adjusted explanatory model controlling for anthropometric and sex-related differences.

Another limitation is that biological maturation status was not assessed. This is particularly important in adolescent athletes because differences in maturation may influence body mass, strength development, coordination, acceleration capacity, and segmental movement patterns. Therefore, future studies should include maturity-related indicators, such as maturity offset, peak height velocity, Tanner stage, or other validated approaches, to better control interindividual developmental differences in young speed skaters.

A further limitation concerns the video sampling frequency. Although 60 fps allowed the analysis of overall segmental motion during the complete 20 m start phase, this frequency is lower than the 120–240 fps commonly recommended for detailed analysis of high-speed sports movements. Consequently, the possibility of motion blur, tracking error, and reduced precision in the estimation of velocity and acceleration derivatives cannot be ruled out. Future studies should use high-speed cameras with higher frame rates and controlled shutter speed to improve the accuracy of segmental tracking and the estimation of rapid kinematic events.

Finally, the estimation of body-segment kinematic variables through video analysis represents an innovative methodological approach in skating. Future studies should integrate physiological variables with biomechanical indicators to provide a more comprehensive characterization of athlete performance and a better understanding of the factors associated with speed skating performance.

## 5. Conclusions

In this exploratory analysis, the forearm segmental inertial force estimate, thigh velocity, and thigh angular velocity were associated with shorter 20 m start times, whereas forearm velocity was associated with longer times. Torso angular acceleration also showed a negative association with start time, although this estimate was less stable in the bootstrap analysis and should therefore be interpreted cautiously. These findings represent candidate biomechanical associations identified in a small dataset evaluated through internal cross-validation and should not be interpreted as stable or causal determinants of performance.

From an applied perspective, the identified variables may serve primarily as provisional indicators for performance monitoring and as a basis for generating testable hypotheses about the biomechanical organization of the start phase. They should not yet be used as direct targets for immediate training optimization or prescriptive intervention or as criteria for athlete selection, talent identification, ranking, or exclusion because movement coordination was not measured directly, the force-related variables were indirect segmental inertial estimates derived from two-dimensional kinematics, measurement reliability was not formally quantified, and external validation was not performed.

Replication in larger independent cohorts, together with formal reliability assessment, three-dimensional or direct kinetic measurements, integration of relevant physiological variables, and external predictive validation, is required to determine whether these candidate variables are reproducible, generalizable, and sufficiently robust to inform individualized training decisions.

## Figures and Tables

**Table 1:** Descriptive statistics for the segmental biomechanical variables of the upper limb estimated using Tracker and the segment method.

Variable	Mean	Standard Deviation	Min.	Max.
20_meters	3.48	0.17	3.12	3.93
bodyweight	56.76	8.33	47.95	81.00
arm_vel	0.49	0.10	0.39	0.74
arm_accel	2.74	2.04	0.90	7.87
arm_angl	41.90	3.28	36.59	47.31
arm_angul_vel	7.93	1.46	5.61	10.65
arm_angul_accel	3.52	1.36	1.60	6.75
arm _inertial_for_estimat	1.49	0.26	1.23	2.20
arm_pow	0.73	0.18	0.57	1.10
forearm_vel	0.59	0.13	0.41	0.83
forearm_accel	3.02	1.87	0.98	7.59
forearm_angl	39.06	3.68	32.80	44.18
forearm_angul_vel	8.41	1.94	5.38	11.01
forearm_angul_accel	4.50	2.87	0.73	11.14

Note: arm_vel = arm velocity; arm_accel = arm acceleration; arm_angl = arm angle; arm_angul_vel = arm angular velocity; arm_angul_accel = arm angular acceleration; arm_inertial_for_estimat = arm segmental inertial force estimate; arm_pow = arm segmental inertial power estimate; forearm_vel = forearm velocity; forearm_accel = forearm acceleration; forearm_angl = forearm angle; forearm_angul_vel = forearm angular velocity; forearm_angul_accel = forearm angular acceleration. Std = standard deviation; Min = minimum; Max = maximum.

**Table 2:** Results of the heteroskedasticity-robust linear regression model (HC3) for predicting 20 m start time.

Parameter	β	se_HC3	t	*p*_val	95CI_Low	95CI_High
const	4.7	0.18	26	1.1e-15	4.4	5.1
forearm_inertial_for_estimat	−1.0	0.13	−7.9	3.0 × 10^−7^	−1.3	−0.74
thigh_vel	−1.9	0.53	−3.7	0.0017	−3.0	−0.84
forearm_vel	1.4	0.37	3.9	0.00095	0.67	2.2
thigh_angul_vel	−0.034	0.0059	−5.7	2.2 × 10^−5^	−0.046	−0.021
torso_angul_accel	−0.030	0.0097	−3.1	0.0069	−0.050	−0.0092

Note: Const = constant; forearm _inertial_for_estimat = forearm segmental inertial force estimate; thigh_vel = thigh velocity; forearm_vel = forearm velocity; thigh_angul_vel = thigh angular velocity; torso_angul_accel = torso angular acceleration; β = unstandardized regression coefficient expressed in the original units of the predictors and outcome; se_HC3 = robust standard error; t = t-statistic; *p*_val = *p*-value; 95CI_low = lower limit of the 95% confidence interval; 95CI_high = upper limit of the 95% confidence interval. The *p*_val values are reported as descriptive post-selection statistics and should not be interpreted as conventional confirmatory inferential *p*-values because the variables were selected using a data-driven LASSO procedure before fitting the OLS model.

## Data Availability

Data are not publicly available due to the sensitive nature of the information and confidentiality considerations. The data supporting the findings of this study are available from the corresponding author upon reasonable request, subject to approval and compliance with applicable ethical requirements.

## Abbreviations

The following abbreviations are used in this manuscript:

arm_vel	Arm velocity
arm_accel	Arm acceleration
arm_angl	Arm angle
arm_angul_vel	Arm angular velocity
arm_inertial_for_estimat	Arm segmental inertial force estimate
arm_inertial_pow_estimat	Arm segmental inertial power estimate
forearm_vel	Forearm velocity
forearm_accel	Forearm acceleration
forearm_angl	Forearm angle
forearm_angul_vel	Forearm angular velocity
forearm_angul_accel	Forearm angular acceleration
torso_angul_accel	Torso angular acceleration
torso_inertial_for_estimat	Torso segmental inertial force estimate4
thigh_inertial_for_estimat	Thigh segmental inertial force estimate
thigh_angul_vel	Thigh angular velocity
thigh_angular_accel	Thigh angular acceleration
torso_inertial_pow_estimat	Torso segmental inertial power estimate
LOOCV	Leave-one-out cross-validation
LASSO	Least Absolute Shrinkage and Selection Operator

## References

[1] Konings M.J., Elferink-Gemser M.T., Stoter I.K., van der Meer D., Otten E., Hettinga F.J. (2015). Performance characteristics of long-track speed skaters: A literature review. Sports Med..

[2] Chavarro J.G., Peña Y.F., García M.Á.G., Aristizabal D.F.O., Del Cristo Martinez Gutiérrez A. (2026). Efectos causales de variables biomecánicas sobre la velocidad inicial en patinadores de velocidad: Un enfoque bayesiano guiado por diagramas acíclicos dirigidos (DAGs). Retos.

[3] Gómez García M.Á., Florez Peña Y., Gaviria Chavarro J., Orejuela Aristizabal D.F. (2025). Análisis de factores físicos en los primeros 20 metros de la fase de salida en patinaje de velocidad. Retos.

[4] Helesic J., Lehnert M. (2024). Explosive strength and velocity as potential determinants of success in youth figure skating competitions. Appl. Sci..

[5] de Koning J.J., van Ingen Schenau G.J., Zatsiorsky V.M. (2000). Performance-determining factors in speed skating. Biomechanics in Sport: Performance Enhancement and Injury Prevention.

[6] Kimura Y., Yokozawa T. (2024). Skating techniques of ladies’ world-class long-distance speed skaters to shorten curved-section time during the official 3000 m race. Front. Sports Act. Living.

[7] Perez J., Guilhem G., Hager R., Brocherie F. (2021). Mechanical determinants of forward skating sprint inferred from off- and on-ice force-velocity evaluations in elite female ice hockey players. Eur. J. Sport Sci..

[8] Van Den Tillaar R., Pojskic H., Andersson H. (2025). Spatiotemporal analysis of linear skating sprint in male and female bandy players: Analysis of acceleration and maximal velocity phase. Biomechanics.

[9] Kibler W.B., Press J., Sciascia A. (2006). The role of core stability in athletic function. Sports Med..

[10] Battaglia G., Giustino V., Iovane A., Bellafiore M., Martines F., Patti A., Traina M., Messina G., Palma A. (2016). Influence of occlusal vertical dimension on cervical spine mobility in sports subjects. Acta Medica Mediterr..

[11] Yordanova T. (2020). Analysis of the dependence between jumping takeoff and anthropometric indicators of female figure skaters. J. Appl. Sports Sci..

[12] Seeberg T.M., Kocbach J., Danielsen J., Noordhof D.A., Skovereng K., Haugnes P., Tjønnås J., Sandbakk Ø. (2021). Physiological and biomechanical determinants of sprint ability following variable intensity exercise when roller ski skating. Front. Physiol..

[13] Bongiorno G., Sisti G., Dal Mas F., Biancuzzi H., Varrecchia T., Chini G., Ranavolo A., Pellegrini B., Bortolan L., Miceli L. (2024). The kinematic and electromyographic analysis of roller skating at different speeds on a treadmill: A case study. Sensors.

[14] Wu Z., Cardoso F., Pyne D.B., Goethel M.F., Fernandes R.J. (2025). Physiological and biomechanical characteristics of inline speed skating: A systematic scoping review. Appl. Sci..

[15] Park Y., Moon J., Lee E.C. (2023). Automatic stroke measurement method in speed skating: Analysis of the first 100 m after the start. Electronics.

[16] Pipkin A., Kotecki K., Hetzel S., Heiderscheit B. (2016). Reliability of a qualitative video analysis for running. J. Orthop. Sports Phys. Ther..

[17] Serrien B., Goossens M., Baeyens J. (2019). Statistical parametric mapping of biomechanical one-dimensional data with Bayesian inference. Int. Biomech..

[18] Guan C., Ren H., Zhang H., Zhang C. (2024). Human mechanics modeling and experimental research in speed skating sports. J. Mech. Med. Biol..

[19] Wang H., Wu Y., Liu J., Zhu X. (2023). Investigation of the mechanical response of the foot structure considering push-off angles in speed skating. Bioengineering.

[20] Iizuka T., Tomita Y. (2024). Reliability of motion phase identification for long-track speed skating using inertial measurement units. PeerJ.

[21] De Leva P. (1996). Adjustments to Zatsiorsky-Seluyanov’s segment inertia parameters. J. Biomech..

[22] van der Kruk E., Veeger H.E.J., van der Helm F.C.T., Schwab A.L. (2017). Design and verification of a simple 3D dynamic model of speed skating which mimics observed forces and motions. J. Biomech..

[23] Gredin N.V., Bishop D.T., Williams A.M., Broadbent D.P. (2020). The use of contextual priors and kinematic information during anticipation in sport: Toward a Bayesian integration framework. Int. Rev. Sport Exerc. Psychol..

[24] Knox C.L., Comstock R.D. (2006). Video analysis of falls experienced by paediatric iceskaters and roller/inline skaters. Br. J. Sports Med..

[25] Dingenen B., Barton C., Janssen T., Benoit A., Malliaras P. (2018). Test-retest reliability of two-dimensional video analysis during running. Phys. Ther. Sport.

[26] Stenroth L., Vartiainen P., Karjalainen P.A. (2023). Force-velocity profiling in ice hockey skating: Reliability and validity of a simple, low-cost field method. Sports Biomech..

[27] Tomita Y., Iizuka T., Irisawa K., Imura S. (2021). Detection of movement events of long-track speed skating using wearable inertial sensors. Sensors.

[28] Lloyd R.S., Oliver J.L., Faigenbaum A.D., Howard R., De Ste Croix M.B.A., Williams C.A., Best T.M., Alvar B.A., Micheli L.J., Thomas D.P. (2015). Long-term athletic development—Part 1: A pathway for all youth. J. Strength Cond. Res..

[29] Malina R.M., Rogol A.D., Cumming S.P., Coelho-e-Silva M.J., Figueiredo A.J. (2015). Biological maturation of youth athletes: Assessment and implications. Br. J. Sports Med..

[30] Moran J., Sandercock G.R.H., Ramírez-Campillo R., Meylan C., Collison J., Parry D.A. (2018). Age-related variation in male youth athletes’ countermovement jump after plyometric training: A meta-analysis of controlled trials. J. Strength Cond. Res..

[31] Arede J., Fernandes J., Moran J., Norris J., Leite N. (2021). Maturity timing and performance in a youth national basketball team: Do early-maturing players dominate?. Int. J. Sports Sci. Coach..

